# Design and characterization of a magnetic bottle electron spectrometer for time-resolved extreme UV and X-ray photoemission spectroscopy of liquid microjets

**DOI:** 10.1063/4.0000107

**Published:** 2021-06-09

**Authors:** Naoya Kurahashi, Stephan Thürmer, Suet Yi Liu, Yo-ichi Yamamoto, Shutaro Karashima, Atanu Bhattacharya, Yoshihiro Ogi, Takuya Horio, Toshinori Suzuki

**Affiliations:** 1 Department of Chemistry, Graduate School of Science, Kyoto University, Kitashirakawa-Oiwakecho, Sakyo, Kyoto 606-8501, Japan; 2 Molecular Reaction Dynamics Research Team, RIKEN Center for Advanced Photonics, 2–1 Hirosawa, Wako 351-0198, Japan

## Abstract

We describe a magnetic bottle time-of-flight electron spectrometer designed for time-resolved photoemission spectroscopy of a liquid microjet using extreme UV and X-ray radiation. The spectrometer can be easily reconfigured depending on experimental requirements and the energy range of interest. To improve the energy resolution at high electron kinetic energy, a retarding potential can be applied either via a stack of electrodes or retarding mesh grids, and a flight-tube extension can be attached to increase the flight time. A gated electron detector was developed to reject intense parasitic signal from light scattered off the surface of the cylindrically shaped liquid microjet. This detector features a two-stage multiplication with a microchannel plate plus a fast-response scintillator followed by an image-intensified photon detector. The performance of the spectrometer was tested at SPring-8 and SACLA, and time-resolved photoelectron spectra were measured for an ultrafast charge transfer to solvent reaction in an aqueous NaI solution with a 200 nm UV pump pulses from a table-top ultrafast laser and the 5.5 keV hard X-ray probe pulses from SACLA.

## I. INTRODUCTION

A magnetic bottle time-of-flight (MBTOF) spectrometer is, despite its very simple design,^1^ a highly capable device for photoemission (PE) spectroscopy, featuring extremely high electron collection efficiency and simultaneous detection of the full spectral energy range on a per-shot basis. These advantages make the spectrometer a preferable choice over a classical hemispherical electron energy analyzer (HEA) for ultrafast pump-probe photoemission spectroscopy with pulsed light sources. Although the energy resolution (Δ*E*) obtainable with MBTOF spectrometers is usually considerably lower than that with a modern HEA, the energy resolution required for ultrafast PE spectroscopy, especially from liquids, is often relatively low (on the order of 0.1 eV), and thus this disadvantage is reduced. While several designs of MBTOF spectrometers for ultrafast laser pump-probe PE spectroscopy of a liquid microjet have been reported,^2–5^ some specific design considerations are required for employing a MBTOF spectrometer in similar experiments using an X-ray free electron laser (XFEL). Here we report a spectrometer and a gated electron detector designed for experiments at SACLA (SPring-8 Angstrom Compact Free Electron Laser).^6^

In recent years, X-ray free electron lasers have been attracting great attention as ultrashort pulsed light sources in the hard X-ray region (a review comparing each facility can be found in Ref. 7). It is noted, however, that pump-probe PE spectroscopy using XFELs is considerably more challenging than using a common table-top UV laser system. For example, SACLA was operated at only 30–60 Hz at the time of our experiments, which is orders of magnitude lower than the kHz repetition rate of table-top lasers. Other XFELs like PAL,^8^ SwissFEL,^9^ or LCLS,^10^ with the still-common nonsuperconducting accelerator technology have similar low repetition rates of up to 120 Hz; only very recently was this barrier lifted by using superconducting accelerators, like at the European XFEL or the LCLS-II upgrade facilities.^7^ PE spectroscopy needs to avoid the so-called space-charge effect caused by Coulombic repulsion between photoelectrons when the density of simultaneously released electrons is high, which energy shifts and/or broadens the electron kinetic energy (eKE) distribution.^11^ Therefore, although XFELs are capable of producing extremely intense X-ray pulses, their pulse energy must always be attenuated for PE spectroscopy. This inevitably diminishes one of the advantages of XFELs over other X-ray sources; however, the temporal duration of the hard X-ray pulses generated by XFELs is unrivaled and considerably shorter than those from other X-ray sources. To mitigate the difficulty from the low repetition rate, it is crucial to use a MBTOF design with its excellent collection efficiency of up to almost 100% of electrons emitted from a gaseous target and as much as 50% (the half-sphere facing the entrance aperture) of electrons emitted from a liquid microjet. Furthermore, spectroscopy using hard X-ray radiation requires to cope with very high absolute kinetic energies (>100 eV) and a wide energy range of the photoelectrons. It is practically impossible to observe the full energy range with a sufficient energy resolution (Δ*E*) even with the time-of-flight (TOF) method, and it is of great assistance to apply a retardation potential to shift the kinetic energy (*E*) of interest to a lower energy where sufficiently high-energy resolution is obtainable. Previously, Mucke *et al.*^12^ have reported a short (0.6 m) MBTOF spectrometer with a retardation potential for measurements in the energy region around 13 eV, and similar MBTOF spectrometers with retardation potentials have been employed for experiments using extreme UV lasers.^4^ Hikosaka *et al.*^13^ tested MBTOF-based PE spectroscopy in a higher electron energy range up to 700 eV using Ar 2p and He 1s photoemission at UVSOR, and they demonstrated that the resolving power (*E*/Δ*E*) of the spectrometer is improved from 35 to 200 with a retardation potential. For achieving a high energy resolution, an alternative approach is to employ a very long TOF tube;^14–16^ however, this approach often conflicts with the finite experimental space inside the radiation-sealed hutches at XFEL beamlines.

In this paper, we present two design approaches for retarding the incoming electrons, a multistage electrostatic lens system and a mesh array, each with its unique advantages. Both are easily swappable depending on the experimental requirements. Additionally, our design features a drop-in extension which, together with the compact mesh array, more than doubles the field-free flight length of the TOF tube (to up to 1.8^ ^m) for improved energy resolution. Although a greater TOF tube length is expected to improve the energy resolution further, the practical length allowed for the spectrometer was limited by the size of an experimental hutch at SACLA and for reasons of ease of operation. The well-established liquid-microjet technique minimizes the gas-pressure from highly volatile liquids and the fast liquid flow minimizes radiation damage of the sample. However, the chamber pressure on the order of 10^−4 ^Torr must be further reduced to operate the microchannel plate (MCP) detector at a pressure of 10^−6 ^Torr or lower. Differential pumping of the MBTOF spectrometer is achieved by constricting the spectrometer orifice down to about 1–2 mm via a graphite-coated conical skimmer. Because of the cylindrical shape of liquid jets, inevitably some part of the pump and probe light pulses are scattered into the TOF tube toward the detector surface. As the MCP is operated at a relatively high gain to mitigate a low count rate of the electron signal of interest, the scattered light can cause the signal output to exceed reasonable limits which also quenches the detector until the excess charge on the MCP is dissipated, and the MCP may even be permanently damaged. We developed a gated MCP which can be switched off on a per-pulse basis for a freely determined time window. This enables us to maximize the detector gain for the weak electron signal of interest while rejecting other intense signals including the scatter light and the associated background electrons. Our MBTOF design is thus highly flexible and can be utilized for a wide range of experimental applications in the extreme UV (XUV) to hard X-ray energy range.

## II. APPARATUS

### A. MBTOF

The principle of an MBTOF spectrometer has been described in detail by Kruit and Read;^1^ contemporary designs usually use a permanent magnet^4,12,13^ instead of the electromagnet described in their paper. The essence of the MB principle is the parallelization of electron trajectories in a magnetic field gradient produced by a combination of a strong and a weak magnet. Electrons are forced into a helical motion around the field lines, and when the variation of the magnetic field gradient from the high to low field region is sufficiently gradual, i.e., adiabatic, then the angular momentum of the electron is conserved. The conservation of the angular momentum reduces the transverse velocity component of the electrons and thus provides parallelization of the electron trajectories in the direction of the flight tube. This favorable condition is quantitatively characterized by the so-called adiabaticity parameter given by^1^

$$\chi =\frac{2\pi mv}{{eB}_{z}^{2}}\left|\frac{dB_{z}}{dz}\right|=2\pi \sqrt{\frac{m}{e}}\frac{\sqrt{2E\left[eV\right]}}{B_{z}^{2}}\left|\frac{dB_{z}}{dz}\right|,$$ (1)

where *m* and *v* are the electron mass and speed, respectively, and *e* is the elementary charge. *E* is the eKE in eV, *B*_z_ is the magnetic field strength, and *z* is the distance from the ionization point along the flight axis. Maintaining an adiabatic condition (small value of χ) requires a low eKE and a high absolute magnetic field while keeping the field gradient along the flight path small.

Our magnetic bottle is formed by a strong magnetic field, created by a permanent magnet that is placed in the vicinity of the ionization point, and a weak homogenous field induced in the solenoid coil inside the TOF analyzer tube. The strong permanent magnet is assembled using a stack of four Sm_2_Co_17_ magnets, each of which is disc-shaped with a diameter of 20 mm and a thickness of 5 mm. A 10-mm long soft iron cone with a half angle of 45° is placed on top of the magnet stack to focus the magnetic flux. The magnetic field strength at the face of the permanent magnet was measured to be 0.44 T, from which the local field strength at the tip of the iron cone was calculated to be 1.60 T using the simulation software Finite Element Method Magnetics (FEMM: www.femm.info). Thus, we estimate the magnetic field strength at the ionization region to be approximately 1 T. Our simulations (see below) indicated that the magnetic field at the ionization point can be increased by 24% by cutting the tip by 1 mm and moving the magnet closer to the ionization point. We employed such a cut cone for hard X-ray experiments. The solenoid coil is made of a silver-plated, PTFE-insulated copper wire with a conductor diameter of 1 mm and shielded with an outer metal shielding layer. The wire was wrapped around the 1.3 m long drift tube (a nonmagnetic stainless-steel tube) with 300 turns. The typical coil current is 3 A, producing a homogenous field strength of 0.9 mT inside the drift tube. After 10 h of operation in vacuum, the coil temperature reaches a plateau of 78 °C. A higher coil current of 10 A would reduce χ to half the value reached when using a coil current of 3 A; however, it would require water cooling of the wire for stable operation.

**FIG. 1.**
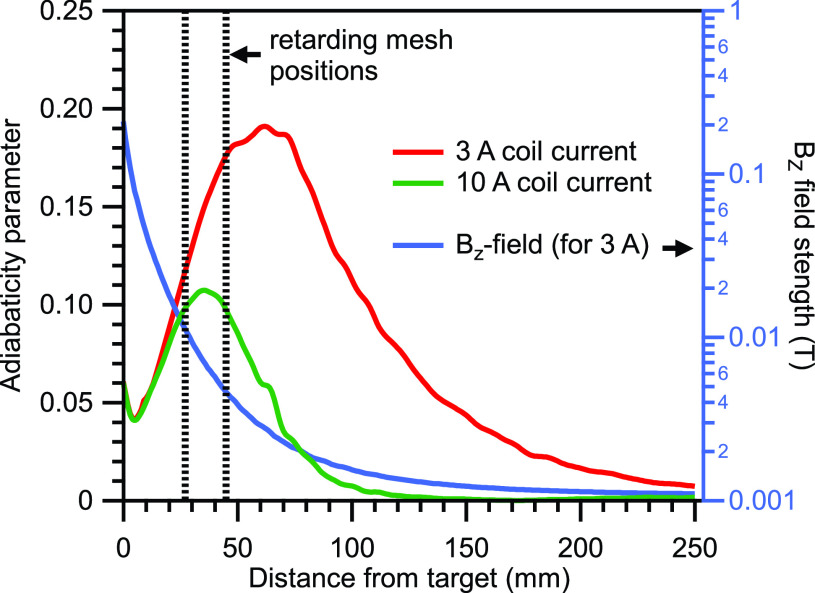
Evolution of the adiabaticity parameter χ (red and green; left scale with values for E = 1 eV) and magnetic field strength along the TOF flight direction B_Z_ (blue; right scale) vs distance from the target (i.e., the liquid-jet nozzle) which is assumed to be placed halfway between the magnet tip and the skimmer orifice. The position of the two retarding meshes are marked with dashed lines.

We employed FEMM to calculate the magnetic fields created by our permanent magnet and solenoid-coil combination (see details of Sec. II B below). The resulting adiabaticity parameter according to Equation (1) and magnetic field *B*_*Z*_ along the center flight axis is plotted in Fig. 1. In the simulation, the magnet-skimmer distance was set to 4 mm, and in Fig. 1, the horizontal axis is plotted starting from the assumed position of the liquid-jet target, which is placed exactly halfway between the magnet tip and skimmer. The simulation indicates that χ of our apparatus increases monotonically from the ionization point up to ∼0.18 (in multiples of *E*^1/2^) at about 60 mm along the flight axis for a coil current of 3 A and then declines slowly. For designing and modifying the apparatus, it is desirable to carry out electron trajectory calculations at high eKEs, where the adiabaticity tends to decline. We imported the calculated magnetic field data into the SIMION 3D software package (Scientific Instrument Services) to carry out electron trajectory calculations. This is achieved by exporting the magnetic field values from FEMM, which then get dynamically loaded via a LUA-script into a magnetic array in SIMION. The magnetic field gradient is insufficient for full parallelization of high-energy electrons, so that a bundle of electron trajectories exhibit focusing and defocusing in a wavy pattern along the flight axis in the field-free drift region (in this paper, we refer to the “field-free drift region” as the part of the electron flight path inside the TOF tube where both the electric potential and the magnetic field strength is constant). When the diameter of this bundle becomes greater than the detector diameter, the electron detection efficiency exhibits oscillation with the kinetic energy as some electrons will inevitably miss the electron detector. With our 20 mm diameter detector described below, the oscillation begins to impact the transmission efficiency at around 300 eV.

We designed the TOF energy analyzer to be able to change its electric potential and the electron pass energy. The variable potential is useful for both decelerating high-energy electrons to increase the adiabaticity as well as the energy resolution and accelerating low-energy electrons to improve their transmission efficiency through the spectrometer; usually, the detection efficiency of low-energy electrons declines below 0.5 eV with MBTOFs. Therefore, when we measure slow electrons near zero kinetic energy, we apply at least +0.5 V to the flight tube to increase the electron pass energy through the spectrometer and ensure an uniform detection sensitivity.

### B. Chamber

**FIG. 2.**
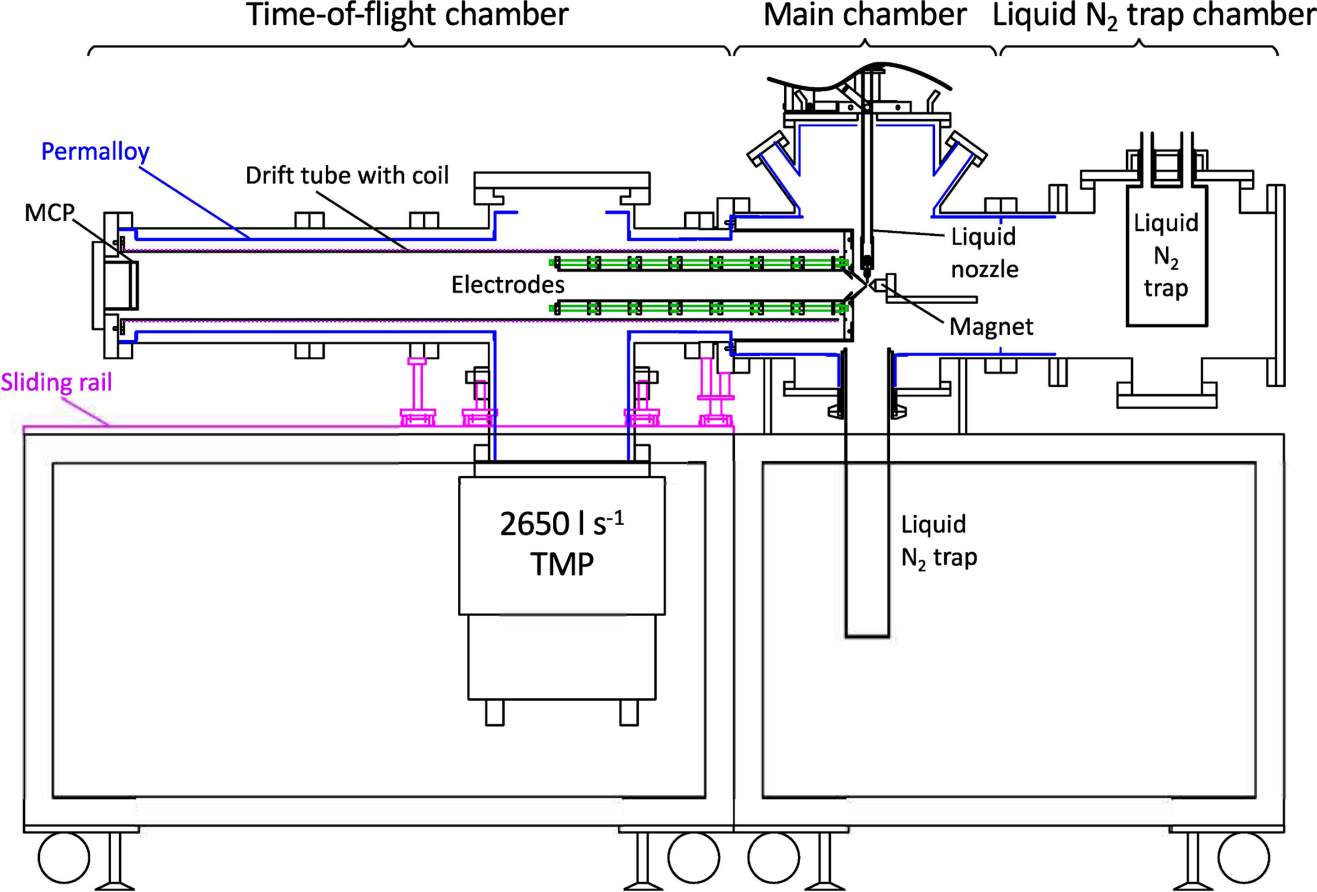
The schematic drawing of our liquid-microjet MBTOF apparatus (side view) equipped here with the standard 1.3-m length TOF photoelectron spectrometer and inserted electrostatic lens system. Indicated in blue is a Permalloy inner layer to shield the apparatus from an external magnetic field.

Figure 2 shows a schematic drawing of our liquid-microjet MBTOF apparatus where the photoelectron spectrometer is configured with a stack of retardation electrodes and features the standard (not elongated) 1.3-m flight tube. It consists of (i) the main chamber (where the laser-liquid interaction occurs), (ii) a liquid-nitrogen trap chamber, and (iii) the TOF electron energy analyzer. The main chamber is evacuated using a turbomolecular pump (TMP, 1400 L/s, Pfeiffer Vacuum, TMU1601P). Pumping of water vapor is strongly boosted by a large (surface area of 1385 cm^2^) liquid-nitrogen-cooled trap; the liquid jet is collected in a second liquid-nitrogen cooled catcher tube below the main chamber. The TOF analyzer is separated from the main chamber with a skimmer orifice of 1–2 mm in diameter and pumped by a TMP with a pumping speed of 2650 l/s (for N_2_ gas, Edwards, STP-XA2703C). The rather large TMP attached to the TOF tube is needed to enable the use of a relatively large skimmer orifice; MBTOF spectrometers designed for UV and EUV radiation usually employ an entrance skimmer of 1 mm diameter. Chang and colleagues employed a 3m long TOF tube and a 0.15 mm pinhole for the electron sampling aperture and achieved *E*/Δ*E* of about 200 without a retarding field, while this sacrificed the electron collection efficiency down to 7%.^16^ On the contrary, our instrument is aimed at a higher electron collection efficiency. Our simulations indicate that a diameter of the entrance skimmer smaller than 1.0 mm clips electron trajectories at an electron kinetic energy higher than 150 eV, while the 2 mm diameter skimmer increases this energy limit up to 300 eV. With the 1 mm skimmer and when running a 25-*μ*m diameter liquid-water microjet at a flow rate of 0.5 ml/min, the pressure in the main chamber and the TOF analyzer are 2.8 × 10^−4 ^Torr and 1.7 × 10^−6 ^Torr, respectively. The entire regions of the main chamber and the TOF analyzer are shielded with a Permalloy inner layer to prevent penetration of the terrestrial magnetic field into the chamber at the photoionization point and the TOF analyzer, which enables linear-drift operation without the magnets. A pair of sliding rails are installed to mechanically support the entire TOF analyzer and to move the device out of the way easily for inspection, servicing and cleaning of the TOF system and main chamber and to install the extension drift tube from the front (see below). For pumping purposes, the drift tube holding the coil of the electromagnet is perforated with holes of Ø4 mm (not shown in the figure).

Liquid samples are injected using a fused silica capillary with a 15–25 *μ*m inner diameter. A constant liquid flow is supplied using a high-pressure liquid chromatography (HPLC) pump (Jasco PU2089) at a typical flow rate of 0.5 ml/min. Laminar flow is observed for about 3–4 mm after ejection from the liquid nozzle orifice and before the liquid beam spontaneously breaks up into a stream of droplets. After traveling a distance of about 110 mm, the liquid is frozen in a liquid-nitrogen-cooled trap with an entrance-aperture diameter of 73 mm. The ionizing laser crosses the laminar flow region within 1 mm downstream from the nozzle. For energy calibration of the spectrometer, a gas sample is leaked into the main chamber via 1/16″ PEEK (polyether ether ketone) tube from a different port. Both the permanent magnet and the liquid jet assemblies are mounted separately on XYZ manipulators for fine positioning relative to the light-interaction point and the entrance of the TOF analyzer (the skimmer). The distance between the skimmer and the magnetic tip was usually adjusted to be 3–4 mm (compare Fig. 6). The vacuum components are graphite coated to avoid any uneven potentials and to make their work functions equal.

### C. Electrostatic lens

**FIG. 3.**
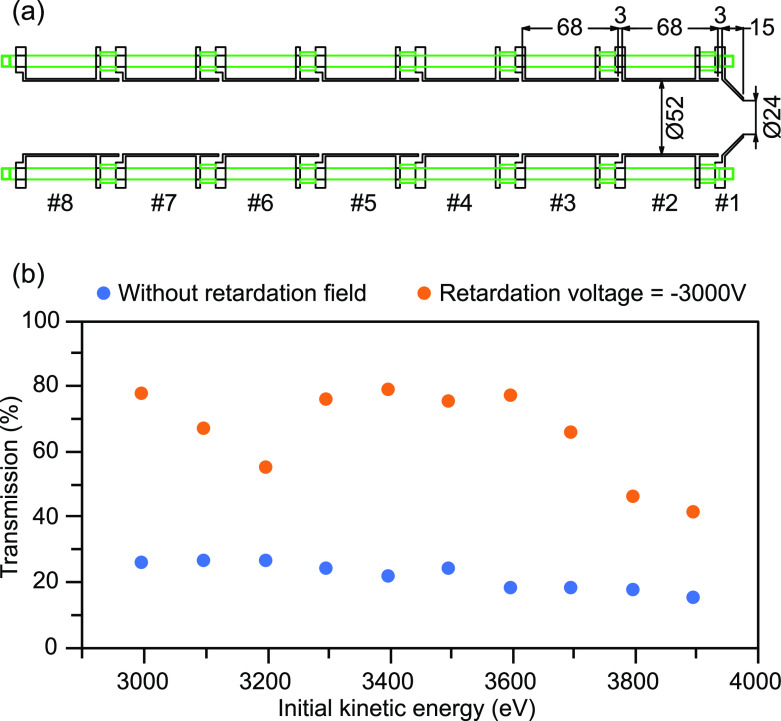
(a) Cross-sectional view of our electrostatic lens system (all units in millimeter). All electrodes were supported on four PEEK insulating rods separated by PEEK insulating spacers (green). (b) Simulated transmission efficiency (percentage of emitted electrons from the target which passes the entire TOF region and hit the ϕ 42 mm MCP) as a function of the initial kinetic energy of electrons with a 2 mm entrance skimmer (blue: with magnetic bottle only; orange: with additional applied retardation voltage to the electrostatic lens system); here, electrons were assumed to be emitted randomly within a cone angle of ±90° (half-sphere) from the sample toward the TOF tube.

Figure 3(a) shows the cross-sectional view of our electrostatic lens system, which is composed of stacked circular aluminum ring electrodes, and PEEK spacers were used to insulate them from one another. For a retardation field of –3000 V, the first eight electrodes [#1–#8 in Fig. 3(a)] have a potential of 63.3% (–1900 V), 83.3% (–2500 V), 66.7% (–2000 V), 0.3% (–8 V), 10.9% (–327 V), 68.2% (–2045 V), 72.7% (–2180 V), 80% (–2400 V) of the total retardation voltage (–3000 V), respectively. The last electrode (the drift tube itself) and the front mesh of the MCP were set to be at equal potential, which is set 100% of the total voltage (–3000 V), to provide a field-free drift region. A screening mesh is placed in front of the MCP assembly in order to prevent leakage of electric potential from the assembly into the field-free drift region. Voltages of the electrodes, the drift tube, and the MCP mesh are independently set using a computer-controlled multiport power supply (MBS, A-1 Electronics; ±12 kV max). The entrance skimmer and the magnet are grounded so that the electric potential gradient created by the electrodes does not affect electron trajectories in the main chamber. Figure 3(b) shows the transmission efficiency simulated with SIMION. It can be seen that our electrostatic lens in addition to the magnetic bottle enhances electron transmission through the flight tube and increases the detection efficiency.

Although not exploited in our experiments at SACLA and SPring-8, a highly useful application of the electrostatic lens is the focusing of electron trajectories onto the active area of the electron detector in PE anisotropy measurements. In such measurements, the magnetic fields in the ionization region and TOF analyzer are turned off and the photoelectron flux is measured as a function of the polarization direction of the ionizing radiation with respect to the electron detection axis. Under these conditions, the detection solid angle is limited by the diameter (42 mm) of the electron detector and its distance (1.3 m) from the ionization point, because a certain number of electrons that pass the entrance aperture of the TOF tube will still miss the electron detector. However, the electrostatic lens inside the TOF tube can focus the electron trajectories onto the detector to increase the detection solid angle, which is ultimately only limited by the solid angle formed by the electron sampling skimmer in front of the TOF tube. We utilized this capability in ultrafast time- and angle-resolved photoemission spectroscopy of liquid samples in our laboratory in the past.^17^

### D. Retarding mesh

**FIG. 4.**
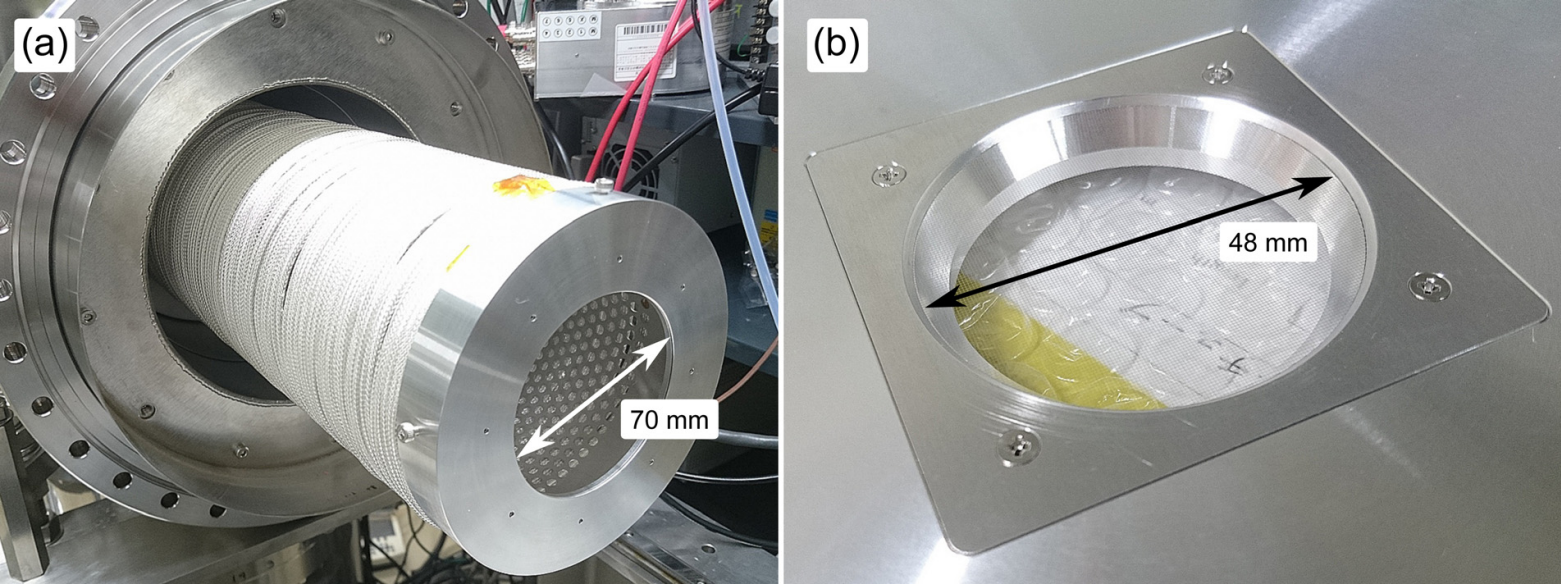
Photos of the retarding meshes: (a) mesh cap and (b) mesh inset. The arrows indicate the diameter of the exposed mesh surface in each case. In the assembled configuration these meshes have a distance of 8 mm (compare Fig. 5). The mesh cap is screwed onto the coil support while the mesh inset is placed into the bulkhead front plate; both are electrically insulated from each other.

**FIG. 5.**
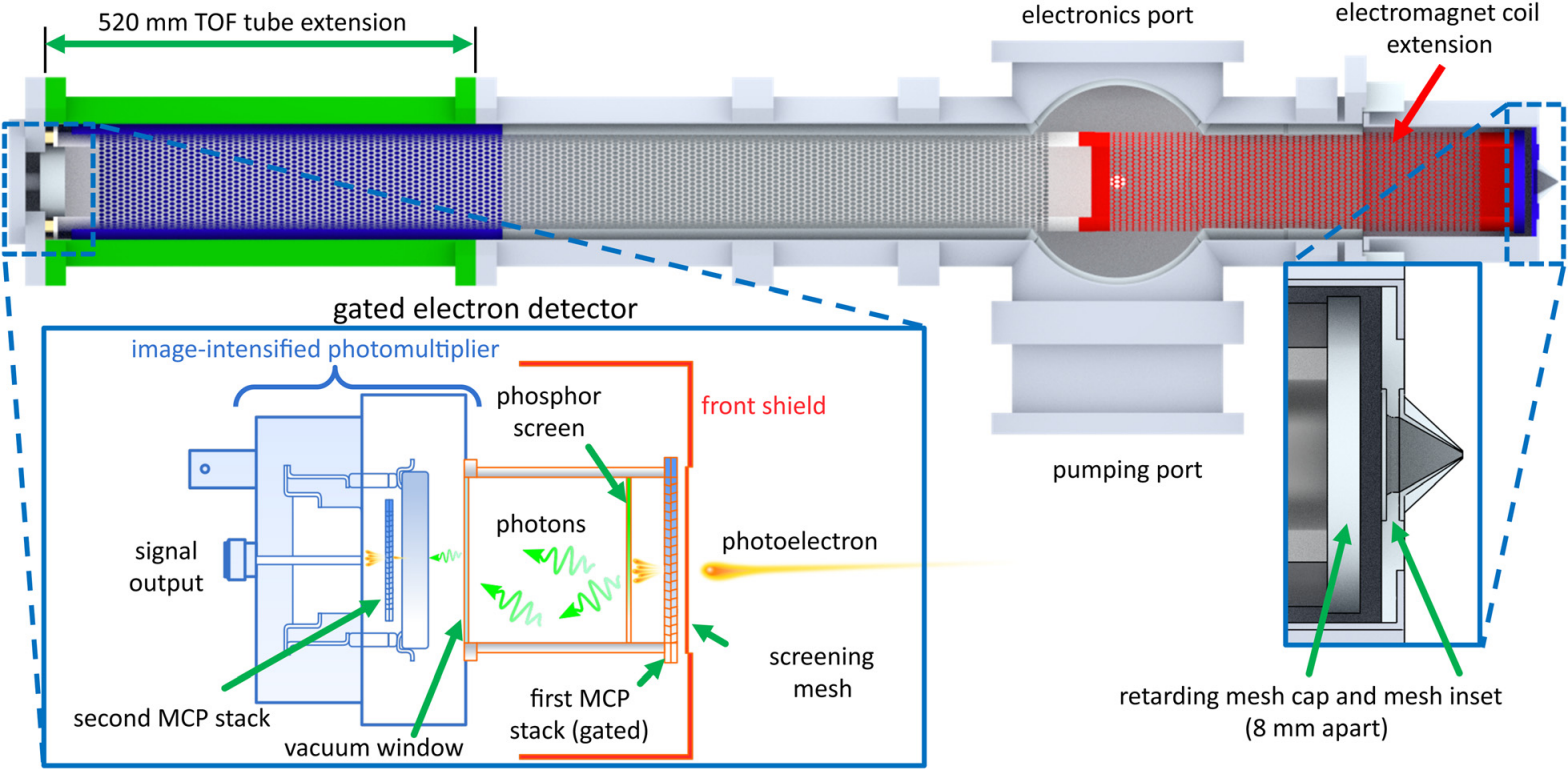
TOF spectrometer in the elongated configuration with the retarding meshes and the gated electron detector. A 520 mm inset brings the total drift tube length to 1763 mm (inner mesh to detector) for improved resolution. The gated electron detector consists of two MCP stacks of which the first can be switched by an external trigger. The conversion to photons in the intermediate step ensures electric decoupling between the first MCP and second MCP (for signal output), thus preventing the introduction of electric noise caused by the gate pulse.

While the electrostatic lens system enables flexible control of the electron pass energy and trajectories depending on the experimental requirements, a drawback is its length of about 50 cm that diminishes the field-free flight length after the lens stack and, consequently, the energy resolution. Therefore, for utilizing the full length of the TOF tube for energy analysis, we replaced the electrostatic lens system with simple retarding meshes (two pieces with 90% open area), similar to those previously employed by Hikosaka *et al.*,^13^ at the entrance of the flight tube behind the skimmer (Fig. 4). We also added an extension to the flight tube to increase the field-free drift region to a total length of about 1.8 m (Fig. 5). In this configuration, trajectory calculations indicate that extreme retardation (more than 93% reduction of the initial kinetic energy) introduces inaccuracies in the measured PE spectra due to band shape distortions; such an extreme condition is, however, never reached in actual measurements. Another drawback of the retardation mesh is that we observed an additional background signal which was absent with the electrostatic lens system described above. We attribute this disturbance signal to secondary photoemission after electron impact onto the retarding mesh material. In principle, this can be mitigated by using meshes with a larger open area ratio.

### E. Gated electron detector

In most of our ultrafast PE-spectroscopic measurements using table-top lasers, we employ a standard chevron-type (dual) MCP detector of 42 mm effective diameter with an electric current readout from the anode in combination with a preamplifier (FAST Comtec, 1.5 GHz) and either a multichannel scaler (FAST Comtec, P7887) with a bin width of 250 ps or an analog-to-digital converter (Acqiris, U1084A-002) for data acquisition. The former is employed at low electron count rates where only one or less than one electron arrives within the response time (several nanoseconds) of the detection system, while the latter is employed at higher count rates. Since the signal amplification in the detector is stochastic, the individual pulse height upon an electron arrival is not uniform; therefore, the multichannel scaler is preferred as long as the count rate is sufficiently low. At SACLA with an operating repetition rate of only 30–60 Hz, the UV pump and hard X-ray probe pulse intensities have to be relatively high. Light scattered off the liquid jet onto the detector surface of the MCP caused an intense signal spike, overwhelming the MCP at the high gain settings needed to detect the desired signal. To circumvent this, a gated electron detector was employed in SACLA experiments. To time-gate an electron detector one needs to switch the high-voltage input for the microchannel plates. However, switching of high voltages causes AC noise in the output current from the MCP anode, which deteriorates the TOF data or even damages the preamplifier circuit. To reject this noise, the signal was electrically isolated from the output by inserting a photoconversion step into the detector assembly, i.e., the signal is converted to light flashes in the first multiplier array and this light signal is then converted back into an electric signal by a second MCP stage. The bottom-left inset of Fig. 5 shows the schematic diagram of our gated detector; only the isolated first MCP stack is switched. We employed a phosphor screen with a short decay time (1–4 ns) to transform electron signals amplified by a MCP (Hamamatsu, F2222–21PF282) to light pulses in the first stage and then observe the light with an image-intensified photomultiplier (Hamamatsu, R3809U-50-MOD) with a response time shorter than 0.3 ns in the second stage. This safely rejected any electric noise from the high-voltage switching of the electron detector. The gate-switching is triggered by a synchronization signal from the light source, which can be additionally offset with a time delay to find the optimal cutout window for the signal. Because of the limited size available for the phosphor screen, the effective diameter of the detector was restricted to 20 mm. An 88-mm-diameter metal plate with a 20-mm-diameter screening mesh in the center was placed in front of MCP to even out the electric potential seen by incoming photoelectrons. The drawbacks of this gated detector are the small effective diameter (only 20 vs 42 mm), being limited by the size of a phosphor screen, and the slower response (at best 1–1.5 ns vs 0.4 ns) as compared to the standard detector.

### F. Alignment

**FIG. 6.**
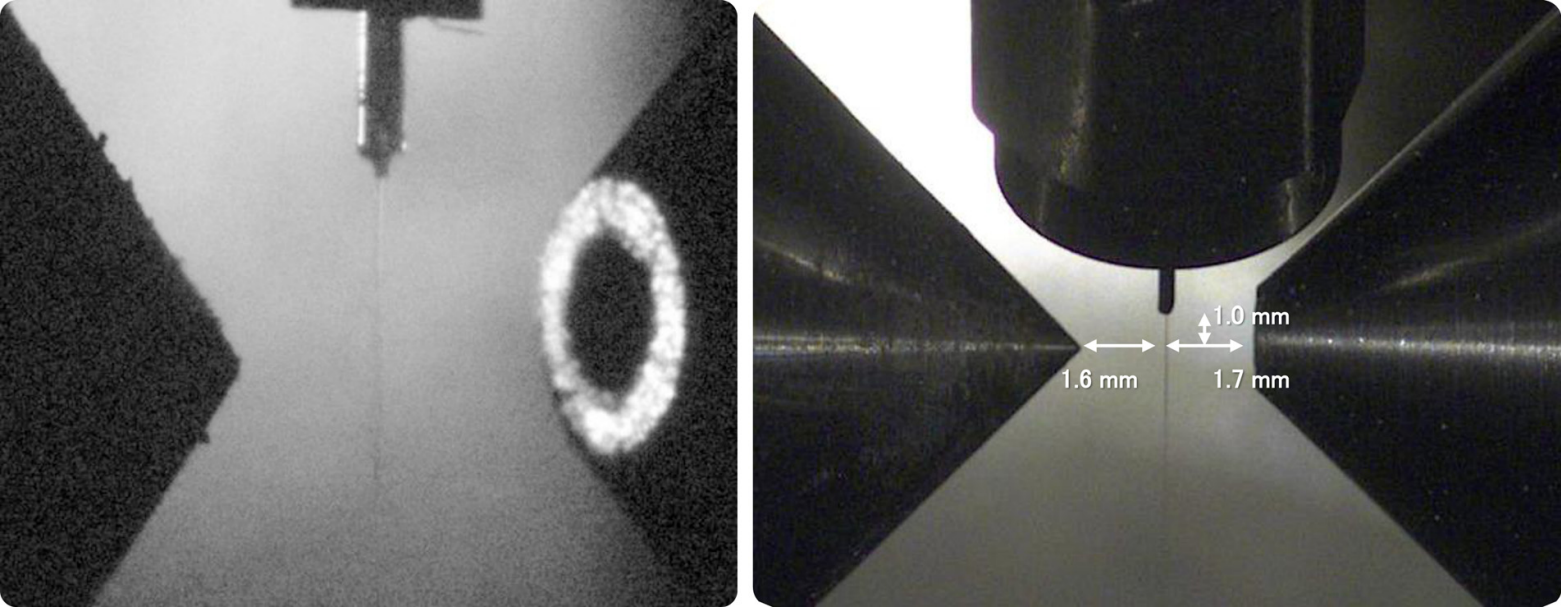
The liquid jet between the permanent magnet (to the left in both images) and skimmer orifice (to the right) is observed from different directions via CCD cameras; here, the side view aligned 135° to the TOF axis (left image) in the horizontal plane and the top view elevated 45° above the X-ray propagation axis (right image) is shown.

Since the propagation direction of the hard X-ray beam at SACLA cannot be manipulated, the entire photoelectron chamber has to be moved in order to bring the X-ray light to the desired interaction position. For this, the entire apparatus was placed on a precision XYZ-translation stage of 1.2 × 2 m^2^ size (AINO Sangyo Co. Ltd.) and moved by computer-controlled stepper motors. For precise alignment of the chamber to the X-ray radiation, we used a thin Ce:YAG rod to visualize the focal spots of the UV and hard X-ray pulses. The image of the Ce:YAG rod was observed via a teleobjective lens through the skimmer orifice from the far end of the TOF tube to precisely center the rod with respect to the TOF tube axis. Then, we illuminated the rod with the UV and the hard X-ray pulses and monitored their focal positions from the rod's fluorescence response with three CCD cameras from different directions. This ensured precise alignment of the deep-UV pump beam to the X-ray beam on the center axis of the TOF tube. Finally, the liquid discharging nozzle was moved to the position marked by Ce:YAG rod, and final adjustments were made by maximizing the actual photoelectron signal. The liquid microjet and permanent magnet positions were adjusted using vacuum-compatible manipulators; a few sample images are shown in Fig. 6.

## III. RESULTS

### A. Estimation of energy resolution

**FIG. 7.**
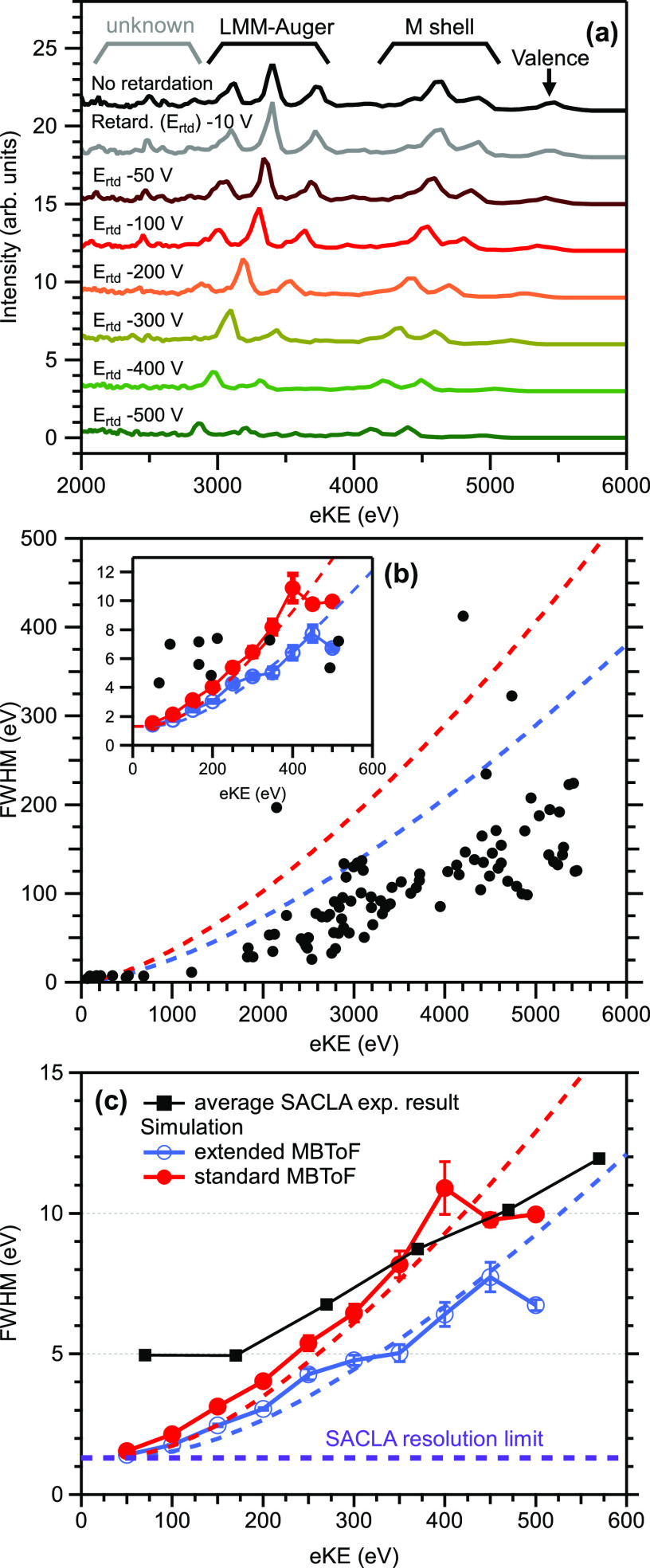
(a) Photoelectron and Auger electron signals measured for Xe with 5.5 keV X-ray radiation and various retardation voltages using the extended (1.8 m) TOF configuration with retarding meshes at SACLA. (b) Comparison of FWHM values achieved for Xe and Ar gases (black) with simulation results (blue dots), and an approximation (red curve) for the extended TOF design. The inset is an enlarged view of the vicinity of the origin. (c) Comparison of average FWHM values achieved for Kr gas at an earlier SACLA experiment using the standard (1.3 m) TOF configuration and electrostatic lens system with simulation results of the standard (red) and extended [blue, same as in (b)] TOF configurations using a mesh; the dashed lines are again an approximation. The extended TOF design achieves a considerable improvement in resolution.

The kinetic energy resolution of our spectrometer was examined using PE spectroscopy of gaseous Ar and Xe with hard X-rays (5.5 keV) from SACLA; here, the configuration of the extended 1.8-m flight tube and the retarding meshes were employed. For Ar, valence (eBE =15.8 eV), KLL Auger (eKE = 2660.5 eV), and K(1s) photoemission (eBE = 3205.9 eV) were measured, while for Xe, valence (eBE = 12.1 eV), M shell (eBE = 676.4 – 1148.7 eV), LMM Auger (eKE = 2500–4000 eV) photoemission were examined.^18,19^ Figure 7(a) shows the photoelectron spectra of Xe shifting with the applied retardation voltage; it is noted that each peak seen in these spectra are comprised not necessarily of a single line but quite possibly an unresolved cluster of lines. The multiple peaks observed below an eKE of 2700 eV do not originate from photoelectron or Auger emission from Xe and are of unclear origin. The peaks shift 1:1 to lower eKEs with increased retardation voltage, while at the same time the peak's FWHM decreases. The intensity appears to decrease with increased retardation voltage, which is ascribed to a nonuniform transmission efficiency at these high energies. As we explained in the Methods section, a part of electron flux misses the electron detector owing to wavy oscillations of electron trajectories when the kinetic energy in the flight tube exceeds 300 eV; thus, the detection efficiency varies with the electron pass energy in the tube. The retardation potential changes the electron pass energy in the TOF tube, which may then lead to a somewhat reduced probability for an electron to hit the detector. Figure 7(b) shows the FWHMs of the measured gas peaks along with the simulated values for the extended TOF tube configuration in blue. In red the theoretical resolution is shown, calculated by using $\Delta E_{\;\text{exp}\;}=\sqrt{\Delta E_{\mathrm{TOF}}^{2}+\Delta E_{\mathrm{SACLA}}^{2}}$ with

$$\Delta E_{\mathrm{TOF}}=\frac{2\Delta t}{L\sqrt{m/2e}}E^{\frac{3}{2}},$$ (2)

where Δ*t* is the overall time-resolution of our instrument (assuming an average of ∼1.25 ns here), *L* is the length of the electron path, and Δ*E*_SACLA_ is the energy resolution of the SACLA beamline (assuming 1.3 eV here). The measured FWHMs are in good agreement with and even smaller than the values predicted using the simple approximation above. Figure 7(c) compares experimental results for the configuration with the standard 1.3-m flight tube length and the electrostatic lens system in comparison with simulations of both the standard and extended flight-tube lengths. Again, the experiment is in good agreement with the simulation, and it can be seen that the flight-tube extension leads to a significant increase in resolution at higher eKEs. For obtaining an energy resolution sufficient to detect chemical shifts on the order of several eV, the absolute value of the kinetic energy needs to be retarded to be less than 300 eV.

### B. X-ray photoelectron spectra

**FIG. 8.**
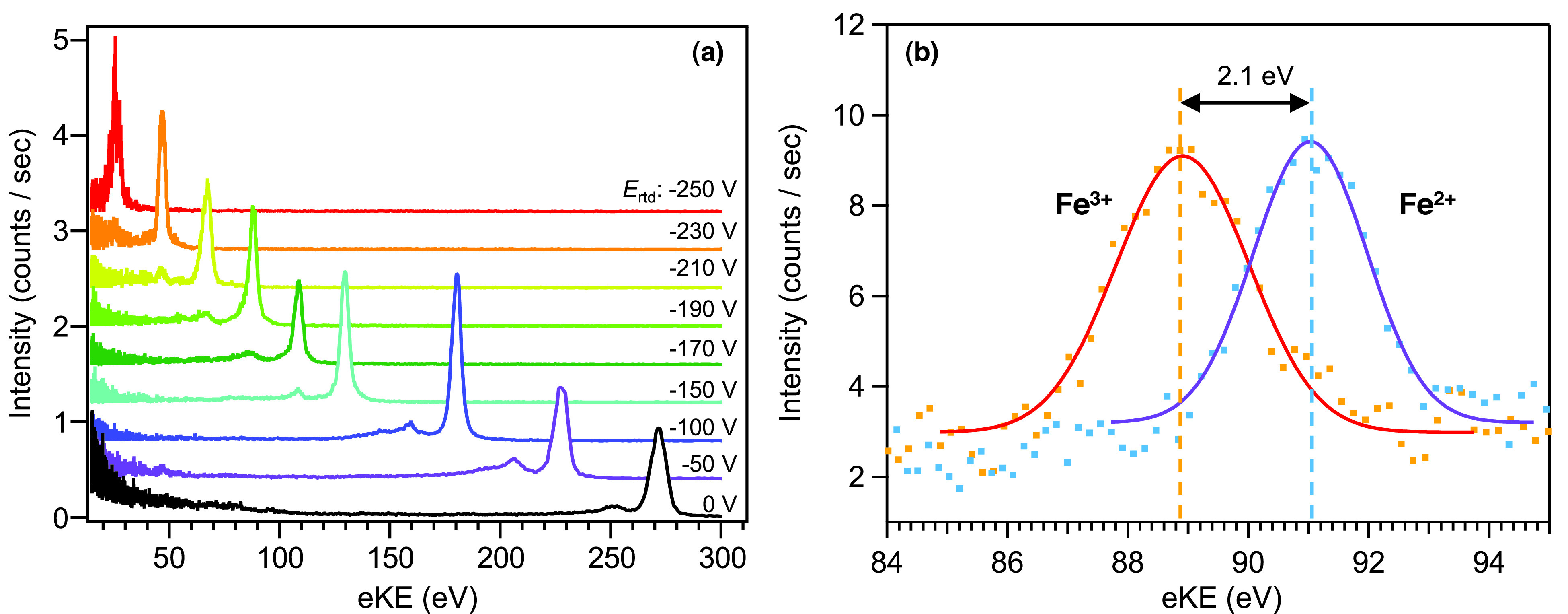
Representative spectra taken at the BL29XU beamline of SPring-8. (a) Kr gas measured at 14.6 keV with retardation voltages between 0 V and –250 V. The retardation bias is translated 1:1 into a PKE reduction, while the resolution increases (lower FWHM). (b) 0.2 M iron hexacyanide aqueous solutions measured at 7.3 keV and with –100 V retardation bias voltage. Both species are spectrally separable with the reached resolution of the device.

**FIG. 9.**
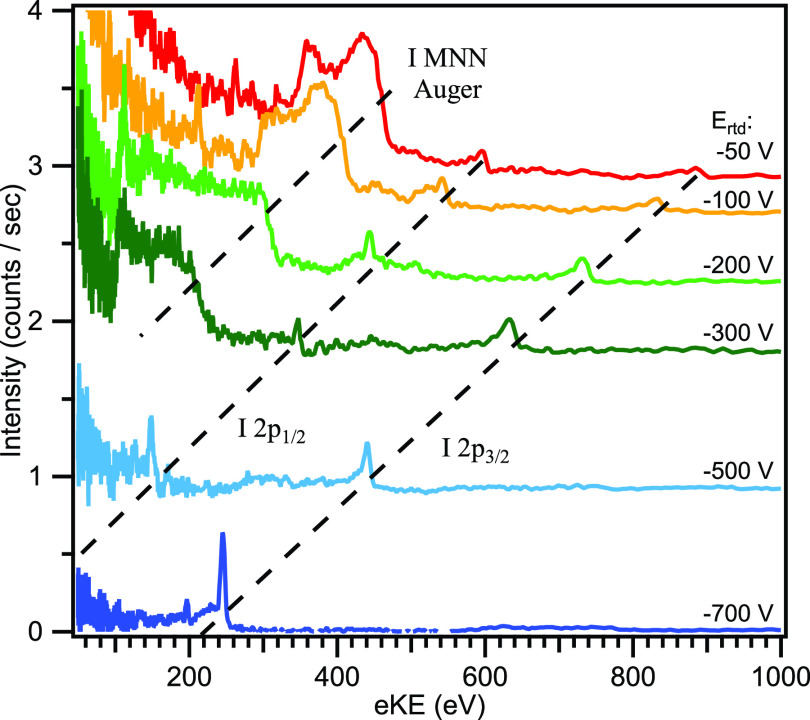
L-shell spectra of 10 mM TBAI (tetrabutylammonium iodide) aqueous solution measured with 5.5 keV and retardation voltages between –50 and –700 V.

Prior to the experiments at SACLA, we performed PE spectroscopy of both gaseous and liquid samples at the BL29 beamline of SPring-8 using the so-called H-mode, and we tested the performance of our MBTOF spectrometer, the alignment method of hard X-ray synchrotron radiation, and the measurement procedure. The H-mode of SPring-8 provides a single bunch at 208.9 kHz and 11/29 (≈ 38% of the remaining ring) filled with a distribution of bunches; the former was used for our measurements. The single-bunch X-ray pulse arrives with a time delay of 1487 ns before/after the continuous 11/29-bunch train (i.e., there is a time window of 2974 ns containing only the single bunch in its center). Figure 8(a) shows a retardation test using the signal from photoionization of Kr gas, which demonstrates the feasibility and usefulness of electron retardation with meshes for the SACLA experiments (see Sec. I.). Then, PE spectroscopy was performed on Fe(1s) photoemission with 7.3 keV photons using 0.2 M aqueous solutions of K_3_[Fe(CN)_6_] and K_4_[Fe(CN)_6_] introduced as 25-*μ*m diameter liquid microjets. Figure 8(b) shows the Fe(1s) signals of both solutions measured using a retardation voltage of –100 V. The Fe(1s) photoemission signals were observed at 88.9 eV (FWHM 2.6 ± 0.1 eV) for Fe^3+^ and at 91.0 eV (FWHM 2.2 ± 0.1 eV) for Fe^2+^, i.e., exhibiting a chemical shift of 2.1 eV. This shift is in reasonable agreement with the values previously estimated from X-ray absorption fine-structure spectra by Bianconi *et al.* (1 eV)^20^ and by Reinhard *et al.* (2 eV).^21^ This demonstrated that the resolution is high enough to spectrally separate these two species with our apparatus and can thus be used in the future for pump-probe experiments to study ultrafast Fe^3+^ ↔ Fe^2+^ charge-transfer processes. Figure 9 shows similar L-shell spectra of iodine measured from 10 mM TBAI (Tetrabutylammonium iodide) aqueous solution with 5.5 keV photons and retardation voltages between –50 and –700 V. PE peaks close to 1 keV were observed, and the energy resolution was improved as retardation is increased (lower voltage).

**FIG. 10.**
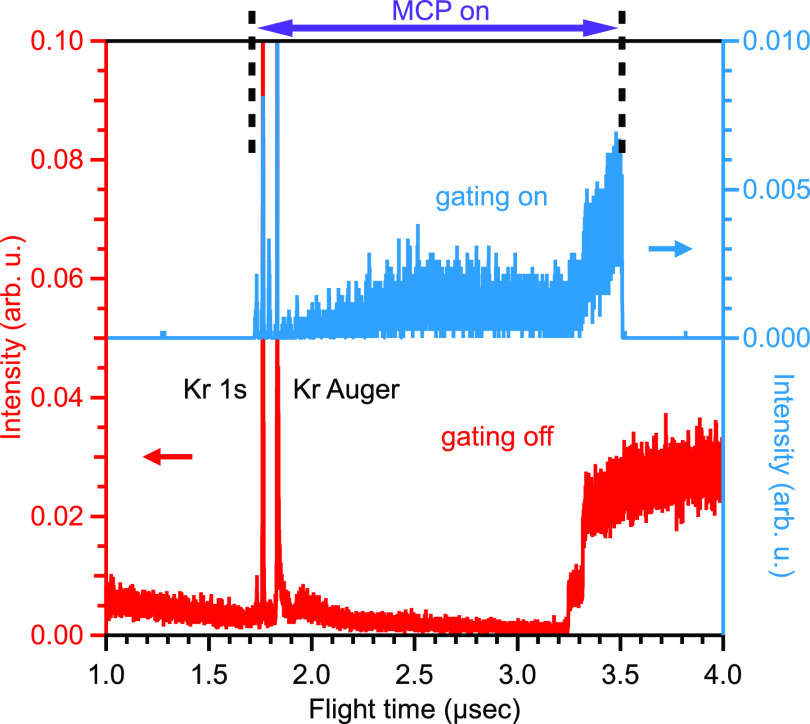
Comparison of photoelectron time-of-flight spectra of Kr gas measured with 14.6 keV photon energy at BL29 beamline of SPring-8 when the MCP gate is activated (blue line; right intensity scale) and deactivated (red line; left intensity scale). The signal drops to zero in the time window where the gating is active. The gating at the maximum frequency of 1 kHz utilizes only 1/209 of the total photon flux of the H-mode at Spring-8, leading to a vastly reduced overall signal (see the text for details).

MCP gating was tested using photoelectrons from Kr, where the continuous light from the 11/29 bunches following the single bunch in the H-mode generates a large photoelectron background (see Fig. 10). By activating the MCP gate, which was triggered in synchronization with the storage ring's acceleration cavity, it was possible to precisely reject a freely selectable part of the photoelectron signal from the multibunch train. As a result, almost no photoelectron signals were observed while the MCP was turned off. At Spring-8, the gating significantly reduced the overall signal intensity (see larger noise floor in Fig. 10) since the gating trigger stage is limited to 1 kHz (optimized for the low repetition rate of SACLA), which thus only utilizes 1/209 of the total intensity from the single pulse repeating with 209 kHz. We also noticed residual trigger noise when the MCP was turned back on, leading to parasitic spikes in the spectrum; no trigger spikes were observed without the gating. Since these noise peaks did not shift even when a retardation voltage was applied, it was easy to distinguish them from true photoelectron signals and remove their contribution.

### C. UV pump and hard X-ray probe experiment

**FIG. 11.**
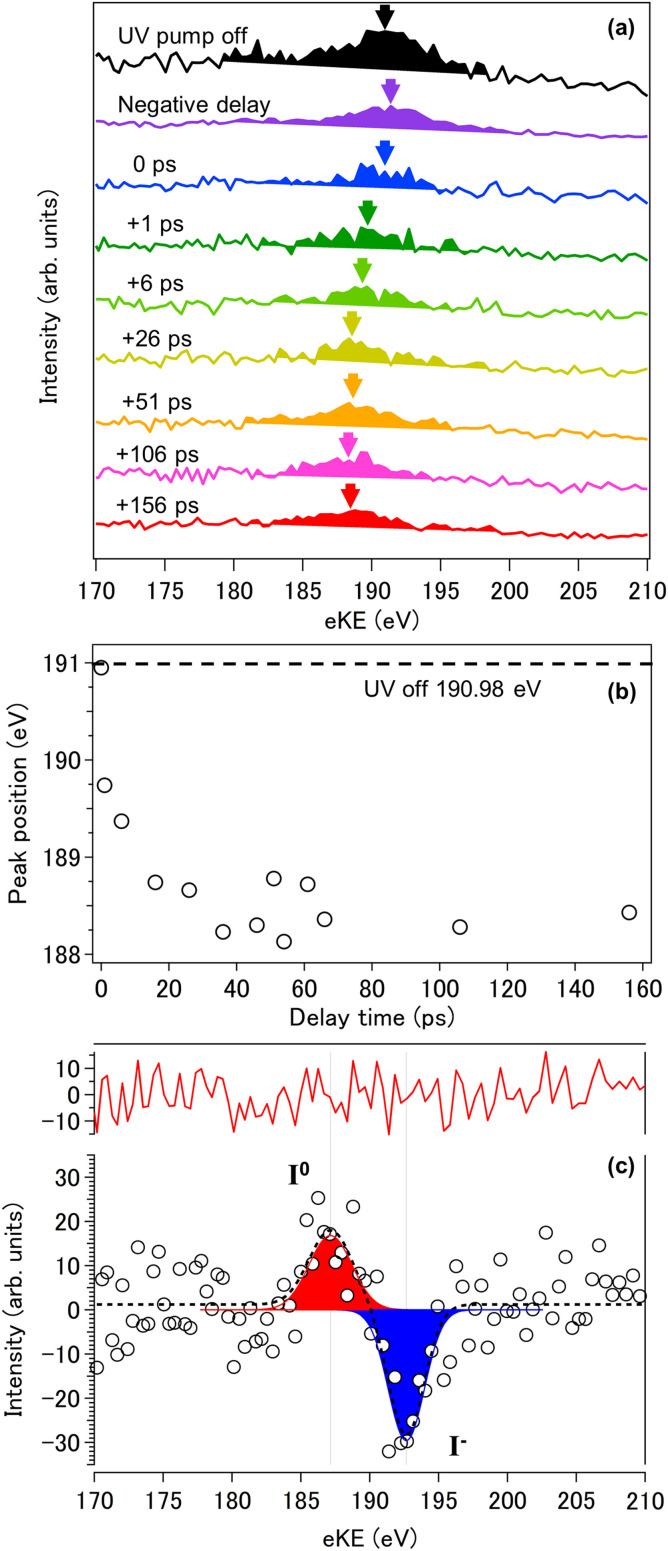
Time-resolved photoemission spectra of the iodine L_3_ shell measured for 0.5 M NaI aqueous solution with 200 nm pump and 5.5 keV probe pulses. (a) Photoelectron spectra measured up to a pump-probe delay time of 156 ps. Arrows indicate the peak center determined using least squares fitting with a Gaussian. (b) Time dependence of photoelectron kinetic energy of iodine L_3_ photoemission features. The dashed line shows the energy observed for I^−^ in the absence of the UV pump pulse. The photoelectron kinetic energy is red-shifted up to a delay time of about 30 ps and then remains almost constant up to 156 ps. (c) Difference spectrum obtained by subtracting the spectrum with negative delay time from the spectrum at 61 ps delay time. The white circles are the measurement results, the dotted lines are the results of multipeak fitting of two components, and the solid red line shows the residuals of the fitting. The negative signal (blue) is ascribed to I^−^ depleted by UV excitation and the positive component (red) is ascribed to neutral I produced by photodetachment.

Finally, we performed UV pump and hard X-ray probe PE spectroscopy at SACLA using a synchronized femtosecond laser system. The pump-probe photoemission spectra measured for a 15-*μ*m diameter liquid microjet of 0.5 M NaI aqueous solution are shown in Fig. 11. The spot sizes of the UV (200 nm, 5.0 *μ*J) and the hard X-ray (5.5 keV, 0.1 mJ) light were 200 × 100 *μ*m and 16 × 24 *μ*m, respectively. The UV spot size was made intentionally larger than that of the hard X-rays to mitigate unavoidable pointing instabilities of the UV beam, which is delivered from a laser system placed outside of the experimental hutch. The UV pump pulses promote a valence electron of I^−^ to a metastable CTTS (charge transfer to solvent) state, which spontaneously decays by detaching the electron to bulk water.^3,22–27^ The aqueous I^−^ solution exhibits two CTTS absorption bands corresponding to I(^2^P_3/2_) and I(^2^P_1/2_) fine-structure levels, and the absorption at 200 nm is resonant with the transition to the CTTS(^2^P_1/2_) band; however, UV photoexcitation at 200 nm produces I(^2^P_3/2_) and an excess electron within 300 fs and no signature of I(^2^P_1/2_) has been observed.^28^ A great number of studies have been performed on this CTTS reaction primarily by observation of the excess electron, while spectroscopic study on the electronic structure of the iodine atom has been scarce; a few exceptional examples are picosecond X-ray absorption spectroscopy using the L_1_ and L_3_ edges of iodine by Chergui and coworkers.^29,30^ Figure 11(a) shows a series of L_3_ edge photoelectron spectra measured at different delay times as well as in the absence of the pump pulse; the photon energy was 5.5 keV. We performed a least squares fit of these bands using a Gaussian function to find the band center as indicated with arrows in Fig. 11(a); it is seen that the band shifts to lower eKE as a function of delay time. Figure 11(b) plots these shifts as a function of delay time between the pump and probe pulses; the total shift of the PE signature was about 2.8 eV. Since electron detachment from the CTTS state of I^−^ occurs on a subpicosecond timescale, the initial spectral shift within 1 ps is primarily due to the detachment, and hydration of the I atom that becomes hydrophobic after the detachment is expected to occur within a few picoseconds. Figure 11(c) shows the difference spectra between the spectra measured at 61 ps and negative delay time; the positive and negative signals represent the signal created and depleted by UV excitation, respectively. Thus the positive and negative signals are ascribed to neutral I and I^−^, respectively. The resulting eKE peak positions of these two species are estimated to be 192.7 and 187.2 eV, indicating a chemical shift of 5.5 eV. A slower relaxation process may also be present after 20 ps, although it is not clearly seen here with the limited signal to noise ratio. One possibility is the formation of a molecular complex such as I(H_2_O).^11^ It is also possible that local heating of the solution may play a role^31^ under the strong excitation condition employed for these measurements. Elucidation of the mechanistic detail awaits further experimental and theoretical studies in the future; however, the present study clearly demonstrates the feasibility of time-resolved PES using a synchronized ultrafast laser and a hard X-ray free electron laser with a MBTOF spectrometer and a gated electron detector.

## IV. CONCLUSION

We have designed and tested a versatile and reconfigurable liquid-jet photoelectron spectrometer based on the simple but powerful magnetic bottle time-of-flight principle for use with hard X-rays of the SACLA free electron laser facility. The photoelectrons are retarded either by an electrostatic lens system or a pair of meshes installed at the front of the flight tube to increase the resolution and reject low-energy electron background signals. While the electrostatic lenses allow for focused retardation conditions, the benefit of the meshes is their compactness, which maximizes the field-free drift region for a higher resolution. The flight tube can be additionally extended to a total length of almost 1.8 m for a further boost of resolution at hard X-ray energies. To reject noise from light pulses scattered onto the detector, a gated two-stage multichannel plate was developed, which allows for switching of the electron signal in a precisely selectable interval. The performance and features of the spectrometer were tested using hard X-rays at BL29 of Spring-8 and the SACLA free electron laser. Retardation test measurements of Ar, Kr, and Xe gas as well as 10 mM TBAI aqueous solution with bias voltages of up to –700 V demonstrated the 1:1 translation of the retardation voltage to a reduction of the photoelectron kinetic energy and an increase in resolution. The resolution was in good agreement with simulations and can be lowered down to spectrally separate PE peak features with a distance of only a few electron volts at hard X-ray energies, which was demonstrated by measuring the Fe^3+^ and Fe^2+^ 1 s core peaks of 0.2 M K_3_[Fe(CN)_6_] and K_4_[Fe(CN)_6_] aqueous solutions; a peak separation of 2.1 eV between these two species was determined. We successfully observed the shift of the iodine L_3_ peak feature of 0.5 M NaI aqueous solution with increasing pump-probe delay time in the experiments using 200 nm UV pump in combination with 5.5 keV hard X-ray-probe pulses, which is ascribed to the creation of neutral I from the photoexcitation of I^−^. The present work demonstrates the feasibility of observing ultrafast (subpicosecond) processes in aqueous solutions with time-resolved PES using a synchronized ultrafast laser and a hard X-ray free electron laser.

## Data Availability

The data that support the findings of this study are available from the corresponding author upon reasonable request.
